# Laparoscopic management of traumatic diaphragmatic rupture with herniation of abdominal contents into left hemithorax

**DOI:** 10.1002/ccr3.7385

**Published:** 2023-05-18

**Authors:** Banwari Lal Bairwa, Ayush Anand

**Affiliations:** ^1^ Department of General and Minimal Access Surgery MP Birla Hospital and Research Center Chittorgarh India; ^2^ BP Koirala Institute of Health Sciences Dharan Nepal

**Keywords:** case report, diaphragmatic rupture, traumatic

## Abstract

**Key Clinical Message:**

Early traumatic diaphragmatic rupture diagnosis using radiological investigations and early surgical management is critical to avoid complications.

**Abstract:**

Traumatic diaphragmatic rupture (TDR) is a rare presentation reported following blunt trauma due to road traffic accidents. Our case showed the importance of early diagnosis of TDR using radiological investigations. Also, early surgical management is critical to avoid complications.

Traumatic diaphragmatic rupture (TDR) is an uncommon occurrence and is reported in 0.3%–1.6% of patients with a history of trauma.^1^, ^2^ The major etiology of TDR is blunt trauma in road traffic accidents (RTA).^1^ TDR can have an acute or delayed presentation, with mortality ranging from 1% to 28%.^1^, ^2^, ^3^ It is more common among middle‐aged males, left‐sided in approximately half of the patients, and associated with rib fractures in 60.7% of patients.^2^ The stomach, colon, omentum, and small bowel often herniate into the thoracic cavity.^4^, ^5^ Though chest X‐ray (CXR) is the first line of imaging modality, the sensitivity is low.^1^ Hence, the mainstay of diagnosis is computed tomography (CT) scan imaging.^1^, ^6^

Herein, we present the case of a 28‐year‐old man who presented to ER with traumatic diaphragmatic rupture with herniation of abdominal contents into left hemithorax, multiple rib fracture, right scapular fracture, and left lung collapse following RTA. CXR revealed an elevated left hemidiaphragm with multiple rib fractures (Figure 1). Focused assessment with sonography for trauma was unremarkable. CT chest and abdomen revealed left hydropneumothorax with atelectasis of the ipsilateral lung and intrathoracic herniation of the gastric fundus and the body, splenic flexure of the colon, spleen with part of the pancreatic tail through the defect in left hemidiaphragm (Figure 2). Also, the CT head was normal.

**FIGURE 1:**
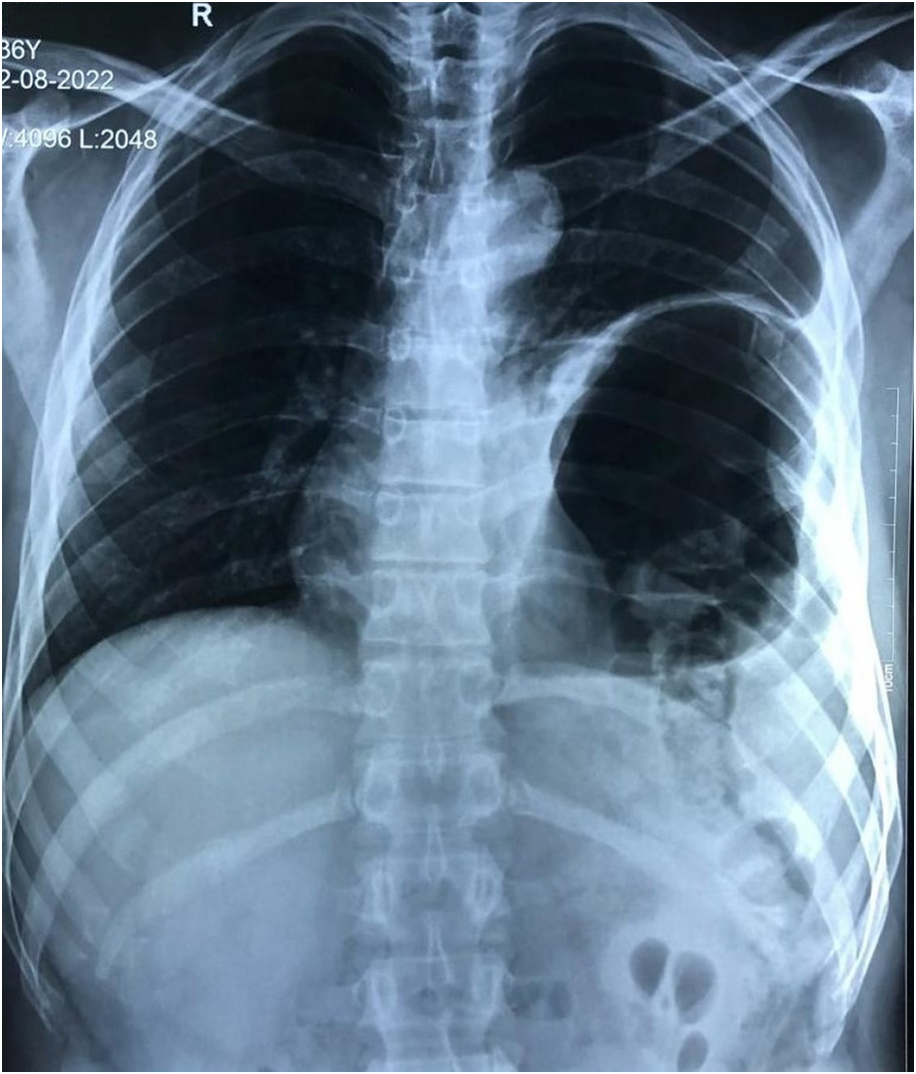
Chest radiograph showing suspected elevation of left hemidiaphragm and air‐filled viscus over the left lower hemithorax.

**FIGURE 2:**
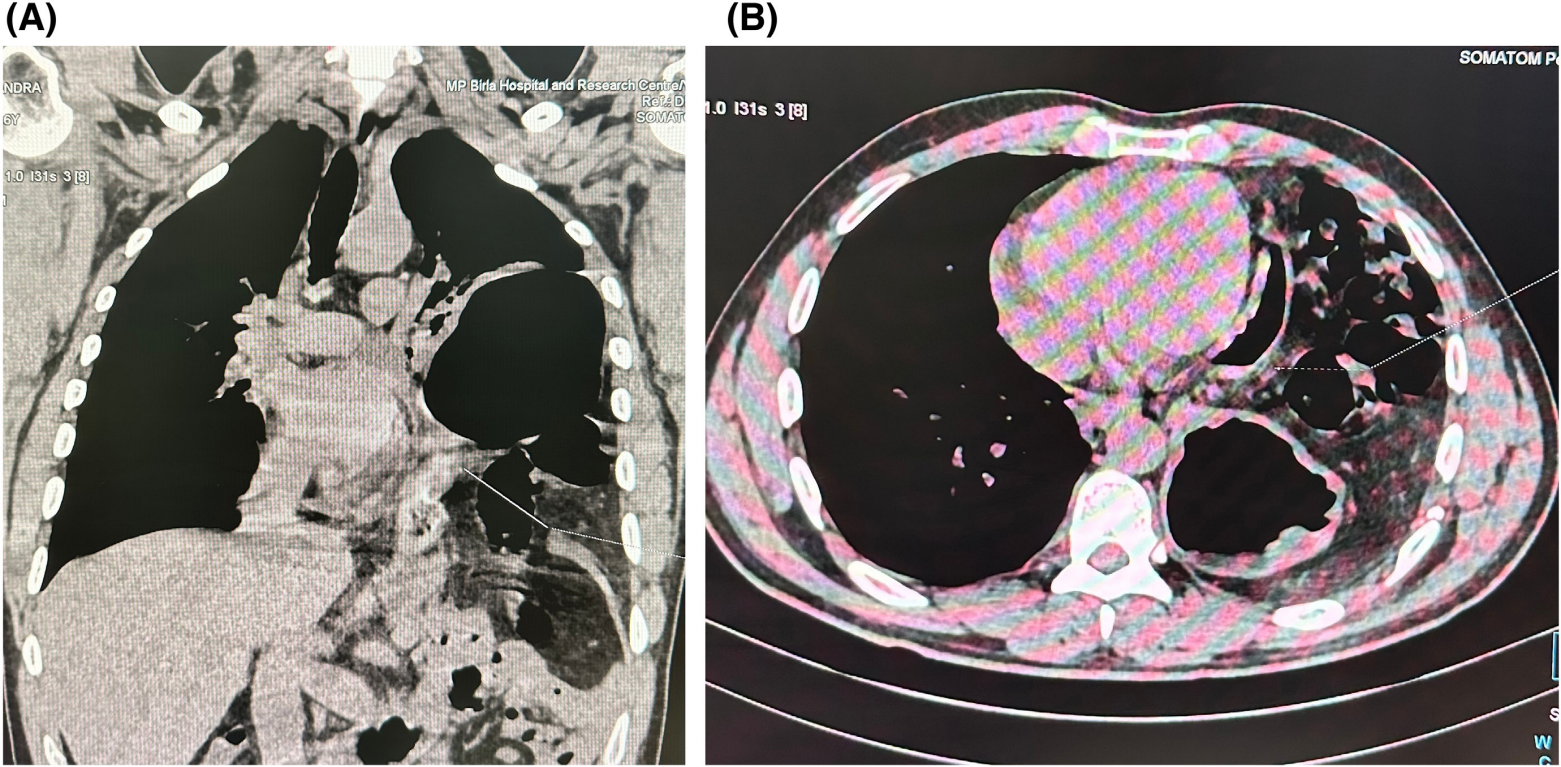
CT chest (A) coronal view and (B) axial view‐ showing left diaphragmatic rupture and herniation of stomach, small bowel, and spleen into left hemithorax.

The mainstay of the management of diaphragmatic rupture is the surgical approach. Silva et al. reported that a higher proportion of patients were treated by an abdominal approach, more so in the acute phase.^7^ The abdominal approach in these patients has a higher chance of intra‐abdominal organ injury.^8^, ^9^ In acute trauma cases, the laparoscopic approach offers better access to survey the abdomen, look for injuries in the thoracic cavity, repair defects, and reposition herniated content.^8^ Studies have also shown that a laparoscopic approach is preferred due to decreased inpatient stay and reduced chances of postoperative complications.^4^, ^7^, ^10^ Hence, we did an emergency exploratory laparoscopy (Figure 3) and placed an intercostal drainage tube (ICDT). The postoperative period was uneventful, and ICDT was removed on the fourth postoperative day. A postoperative CXR revealed minimal pleural effusion with the resolution of initial haziness seen on the initial trauma day chest radiograph (Figure 4). The patient was discharged on the fifth postoperative day with no complications. On follow‐up, after 6 months, the patient was doing well.

**FIGURE 3:**
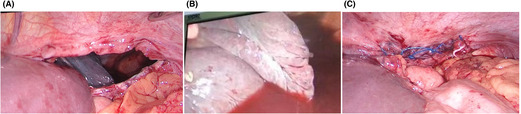
(A) Laparoscopic image showing the diaphragmatic rupture with defect. (B) Laparoscopic image showing hemorrhagic collection in left pleural cavity. (C) Laparoscopic image showing closure of defect with V lock prolene suture.

**FIGURE 4:**
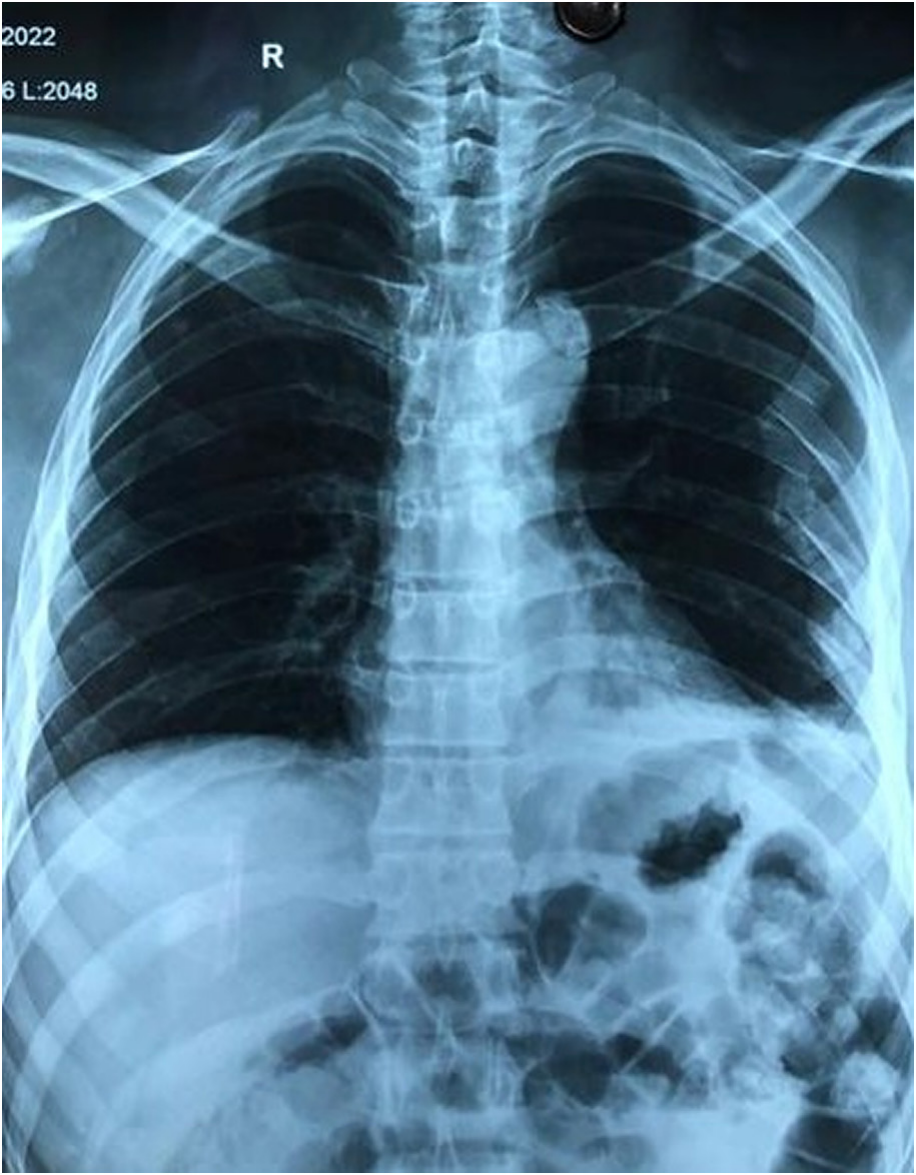
Postoperative chest radiograph showing small pleural effusion and complete resolution of the initial haziness seen on the initial trauma day chest radiograph.

Our case showed that a laparoscopic approach is preferred over an abdominal as a diagnostic and therapeutic tool in acute traumatic cases with diaphragmatic rupture. Its advantages include excellent view and access to the surgical field, faster recovery, and lower postoperative pain with less recurrence rate. In addition, regular follow‐up of patients is crucial to detect recurrences.

## AUTHOR CONTRIBUTIONS

**Banwari Lal Bairwa:** Conceptualization; data curation; investigation; project administration; resources; supervision; validation; visualization; writing – original draft; writing – review and editing. **Ayush Anand:** Conceptualization; data curation; investigation; resources; supervision; validation; visualization; writing – original draft; writing – review and editing.

## FUNDING INFORMATION

The authors did not receive any funding for this manuscript.

## CONFLICT OF INTEREST STATEMENT

The author(s) have no conflict of interests to declare.

## ETHICS STATEMENT

Our institution does not require ethical approval for reporting individual cases or case series.

## CONSENT

Written informed consent was obtained from the patient(s) for their anonymized information to be published in this article.

## Data Availability

All data pertaining to this case are available within this manuscript.
